# Evaluation of a pilot program for task sharing short and long-acting contraceptive methods in Burkina Faso

**DOI:** 10.12688/gatesopenres.13009.2

**Published:** 2020-02-11

**Authors:** Dawn S. Chin-Quee, Kathleen Ridgeway, Yentéma Onadja, Georges Guiella, Guy Martial Bai, Claire Brennan, Georgina Page

**Affiliations:** 1 Department of Global Health Population, and Nutrition, FHI 360, Durham, North Carolina, 27701, USA; 2 Institut Supérieur des Sciences de la Population (ISSP), Ouagadougou, 03 BP 7118 Ouagadougou 03, Burkina Faso; 3 Marie Stopes International, London, London W1T 6LP, UK

**Keywords:** task sharing, community health worker, primary care worker, oral contraceptive pills, injectables, implant, long-acting reversible methods, Burkina Faso

## Abstract

**Background: **The Family Health Directorate of the Ministry of Health  and Marie Stopes Burkina Faso, with implementing partners, Association Burkinabè pour le Bien-être Familial  and Equilibres & Populations  collaboratively conducted a pilot project in Burkina Faso focused on “increasing access to family planning (FP) services through task-sharing short- and long-acting family planning methods to primary care cadres.” Four cadres of providers  provided intrauterine devices (IUDs) and implants, while community health workers (CHWs)  provided pills and subcutaneous injectables. FHI 360 and the Institut Supérieur des Sciences de la Population  evaluated the project’s impact on method uptake, client satisfaction, safety, acceptability and the feasibility of task sharing.

**Methods: **The evaluation employed FP service statistics on new users and conducted 425 client exit interviews  and 27 in-depth interviews . New FP clients, community representatives, MoH officials, and pilot project-trained FP providers from Dandé and Tougan districts participated in these interviews.

**Results: **Providers, community representatives and government officials all spoke favorably of the pilot project and considered it a boon to women and the communities in which they lived. FP clients were satisfied with their methods and the services they received from their respective providers, and they reported no safety concerns. However, service statistics did not show a clear and steady increase in method uptake for the four methods beyond spikes coinciding with pre-existing free contraceptive weeks.

**Conclusions:**  A scale-up plan for 2020-2022 is in place and will purposefully implement sensitization and demand generation activities to improve FP uptake beyond free contraceptive weeks.

## Introduction

Task sharing has been implemented in the field of family planning (FP) to increase access to contraception--particularly for women in rural and underserved areas ^1^. Task sharing facilitates the provision of health services by lower-level, and often more accessible, providers. Provision of long-acting and permanent methods (LAPMs) by clinical and health officers ^2^; long-acting reversible contraceptives (LARCs) by midwives, auxiliary nurses and some lay health workers ^3– 5^ and injectable contraceptives by community health workers ^6, 7^ are all examples of task sharing. The World Health Organization (WHO) endorses task sharing of family planning services and methods by different health worker cadres under certain circumstances, as the evidence supports the feasibility, safety and effectiveness of the practice ^8, 9^. To date, research has demonstrated increased access to condoms, oral contraceptive pills, injectables, implants and tubal ligation through task sharing ^10^, though more research is needed on the safety and effectiveness of non-physician provision of tubal ligation and vasectomy ^8, 11^.

Programs and pilot studies—conducted in countries with acute provider shortages--are leading the way in expanding the scope of and providing the impetus to modify recommendations for FP task sharing. Burkina Faso, a country affected by shortages of qualified FP health providers, is poised to increase access to short- (pills, injectables) and long-acting methods (implants, intrauterine devices (IUDs) by task sharing their provision with registered nurses, registered birth attendants, auxiliary birth attendants, mobile health workers and community health workers (CHWs). These providers are well-placed to address the higher occurrence of unmet need among women in rural (20.7%) versus urban areas (12.2%) of the country ^12, 13^. As reported in the most recent round of Performance Monitoring and Accountability 2020 (PMA2020) data collection (Round 6: December 2018 – January 2019) ^14^, the modern contraceptive prevalence rate (mCPR) in Burkina Faso for women in union was 30.7%, and for all women, 27.3%. Implants accounted for 44.1% of modern method use among women in union—a decrease from 50.3% in Round 5 ^15^--while the subcutaneous formulation of the injectable, depot-medroxyprogesterone acetate (DMPA-SC), and oral contraceptive pills (OCPs) accounted for about 13.1.% and 12.6%, respectively, of modern method use. Intramuscular injectable (DMPA-IM) and intrauterine device (IUD) use were reported by 18.8% and 4.7%, respectively, of women in union. The mCPR has increased steadily for all women and women in union since the first round of PMA2020 conducted in 2014 ^16^, attesting to the Burkinabè government’s commitment to FP2020 goals. Indeed, in the National Plan of Burkina Faso for Acceleration of Family Planning, 2017–2020 ^17^, the Government set a goal for modern contraceptive use at 32% by 2020. The country is tantalizingly close to the stated goal, but it remains to be seen if promoting measures such as task sharing can further increase the mCPR and close the gap between urban and rural areas.

The Family Health Directorate of the Ministry of Health (MoH/FHD) and Marie Stopes Burkina Faso (MS BF), along with implementing partners Association Burkinabè pour le Bien-être Familial (ABBEF) and Equilibres & Populations (Equipop), collaborated as a Consortium to conduct a pilot project in Burkina Faso with the goal of “increasing access to family planning services through task-sharing short- and long-acting family planning methods to primary care cadres.” The pilot project was designed and implemented under the stewardship of the MoH of Burkina Faso, in partnership with MS BF, ABBEF and Equipop. FHI 360 and the Institut Supérieur des Sciences de la Population (ISSP) evaluated this project’s impact on method uptake, client satisfaction, safety, acceptability and feasibility of task sharing. This paper describes the results of the evaluation.

### The Pilot Intervention

The pilot project was implemented in two rural health districts: Dandé in Hauts-Bassins region and Tougan in Boucle du Mouhoun region, which include rural areas with high unmet need for FP ( 23.8% vs. 17.4% in urban areas) and significant potential to increase access to FP. Events that sensitized the community to FP were held in these districts. The pilot implemented task sharing of short-acting methods to community health workers (CHWs) and long-acting methods to four health cadres lower than doctors and clinical officers: registered nurses, registered birth attendants, auxiliary birth attendants, and mobile health workers. Registered nurses are authorized to provide promotional, preventive, curative and rehabilitative services, while registered birth attendants are authorized to counsel, inform, educate and provide comprehensive care as it relates to reproductive health. Auxiliary birth attendants and mobile health workers assist registered birth attendants and registered nurses, respectively, in their authorized tasks. The four are collectively referred to as primary care cadres in this paper and are distinguished as a group from the lay cadre, CHWs.

In total, 79 primary care providers from 26 public health centers in Dandé and Tougan were trained to provide quality FP counseling at facility level, implant and intrauterine device counseling, insertion and removal of these devices, while 128 CHWs affiliated with those health centers were also trained to provide comprehensive FP counseling, prescribe OCPs safely, administer the subcutaneous formulation of injectable contraceptives (DMPA-SC) and deliver key messages at community events like market days ( Table 1). These activities constituted the capacity-strengthening and demand creation components of the pilot intervention ^18^. CHWs began providing services earlier than both groups of primary care providers--by January 2017 after being trained in November and December 2016. Primary care providers in Dandé began providing services in February 2017 (training January to February 2017), while their counterparts in Tougan initiated service provision in April 2017 (training December 2016 to March 2017). By the end of April 2017, all trained providers in both districts had also received follow-up training and supervision.

**Table 1.** Distribution of trained providers and dates of pilot intervention initiation.

	Dandé	Tougan	Total
Health centers	8	18	26
Primary care cadre providers trained			
Licensed nurses	4	12	16
Registered birth attendants	2	6	8
Auxiliary birth attendants	7	22	29
Mobile health workers	10	16	26
Primary care cadre service provision began	February 2017	April 2017	
CHWs trained	34	94	128
CHW service provision began	January 2017	January 2017	

CHW – community health workers.

## Method

### Overview

The objective of the evaluation was to assess whether task sharing long-acting FP services with primary care cadres and short-acting FP services with CHWs is feasible and can increase uptake of high quality, safe and acceptable FP services in Dandé and Tougan districts. The following indicators guided data collection, analysis and interpretation:

- Perceptions of feasibility and acceptability of method provision as reported by primary care cadres, CHWs and key informants;
- Reports of client satisfaction with (and therefore, acceptability of) methods and services received;
- Client reports of service quality (quality of care);
- Number of injuries or adverse events reported with the provision and use of long-acting and short-acting contraceptive methods (safety);
- Comparison of FP uptake statistics on long-acting and short-acting contraceptive methods before and after pilot initiation (calendar years 2016 and 2017).

A mixed methods descriptive evaluation utilizing service statistics and client exit interviews (quantitative) as well as in-depth interviews (qualitative) was conducted between December 2017 and May 2018—commencing several months after primary care providers began providing LARC methods, and almost a year after trained CHWs began providing pills and injectables. This gave intervention sites sufficient time to provide follow-up supervision and to standardize pilot project procedures before initiation of the external evaluation.

A total of 27 in-depth interviews (IDIs) were conducted with a subset of primary care providers and CHWs, community representatives, as well as district and national level MoH personnel. We conducted 425 client exit interviews with women who received methods from providers trained for the pilot project. Service statistics of FP uptake obtained for Tougan and Dandé districts were analyzed to determine changes in uptake over time.

***The quantitative component: family planning clients and service statistics.*** All users new to their chosen long-acting or short-acting method, and who received FP services from a provider trained for and affiliated with any of the 26 project sites, were eligible and approached to participate in the evaluation. Surveys focused on satisfaction with their chosen method and the services received from their providers. To determine the desired sample size, we estimated the proportion of women who would report that their provider talked to them about the possibility of side effects associated with FP method use (a key indicator of service quality). We used a base estimate of 75% for this indicator, resulting in a minimum sample size of 284 clients to estimate this indicator within 5% with a 95% confidence interval. Based on experience, we assumed that at least 75% of providers would discuss the possibility of side effects, because they were trained and received follow-up supervision for the pilot intervention. We did not anticipate needing to use the more conservative standard, a 50% base estimate.

We obtained service statistics from the MoH on FP uptake before initiation and during implementation of the pilot project intervention (calendar years 2016 and 2017) to allow comparisons of FP uptake before and during the intervention. Dandé’s service statistics covered 11 months of the intervention; Tougan’s statistics covered nine months due to the two-month difference in initiation of the pilot.

***The qualitative component: primary care cadres, community health workers, community representatives and government officials.*** With the assistance of Consortium members, we purposively identified and interviewed pertinent individuals for key informant interviews. Two community representatives from Dandé and two from Tougan along with a total of seven district-level and national-level officials from the MoH ^a^ provided feedback on sociocultural, normative (community representatives) and high-level, administrative perspectives (MoH officials) on FP services.

In-depth interviews were conducted with primary care providers and CHWs to understand their respective experiences and views of task sharing FP services. Via convenience sampling, we selected one each of registered nurses, registered birth attendants, auxiliary birth attendants and mobile health workers from both districts, as well as four CHWs each from Dandé and Tougan districts.

### Evaluation procedures

***Family planning client interviews.*** Data collectors, with assistance from participating primary care providers at public health centers and CHWs in the evaluation catchment areas, identified FP clients who were of reproductive age, adult or emancipated if under 18 years of age (i.e., married), and met the criterion for new contraceptive user (recently initiated an FP method they accepted from an intervention-trained provider between December 2017 and February 2018). At health centers, LARC clients who expressed interest in participating in the evaluation were directed to data collectors posted on-site. In health center catchment areas, CHWs informed eligible acceptors of injectables and OCPs about the evaluation. The name and contact information of CHW clients who were interested in participating were promptly given to data collectors who then contacted clients for an interview within one week to reduce recall bias. Before initiating the interview, data collectors confirmed that each client met eligibility criteria and obtained informed consent.

Surveys were administered in French via electronic tablets, with explanations provided as needed to participants in the local languages (Dioula or Mooré). All data collectors were fluent in the three languages. Family planning clients were interviewed between December 12 ^th^, 2017 and February 4 ^th^, 2018 in designated areas that provided audio, and if possible, visual privacy within evaluation facility catchment areas. Interviewers obtained information on clients’ sociodemographic characteristics, experiences using contraceptive methods, factors in client decision to use their chosen method, interaction with and information provided by the primary care provider or CHW, and client satisfaction with her choice of method and with the provider.

***In-depth interviews.*** Both types of providers, community representatives and government officials were asked for their opinions on task sharing in general, and specifically, how the process functioned in the Consortium’s pilot project. Interviews captured perceptions of demand for FP services and the role task sharing plays in creating demand, perceived challenges and successes of task sharing, the availability of FP stocks/commodities, community acceptance/non-acceptance of task sharing FP services, primary care provider and CHW workload and motivation, appraisal of provider training and supportive supervision related to task sharing, and recommendations for scale-up of task sharing in Burkina Faso.

Interviews were conducted in French or the local language using a hard copy interview guide (see extended data ^10^). Interviews were also audio recorded to produce transcripts. Primary care providers were interviewed between December 11 ^th^ and 21 ^st^, 2017 in a private location within the facility. Interviews with the eight CHWs were conducted between December 14 ^th^ and 24 ^th^, 2017 and were also located in a private area within the evaluation facility. The four community representatives were interviewed between December 17 ^th^ and 19 ^th^, 2017 in their homes, while the government officials were interviewed between January 31 ^st^ and March 19 ^th^, 2018 in their offices.

During the evaluation, access to hard copy and electronic data was granted only to staff at ISSP and FHI 360. Informed consent forms signed by evaluation participants were stored in a separate locked drawer or cabinet. Electronic data were stored in password-protected files. Upon completion of the evaluation, all stored materials were destroyed at ISSP. All electronic data were transferred to FHI 360.

### Data analysis procedures

***Quantitative data.*** Client survey data were cleaned and analyzed in Stata 15 ^19^. Frequencies, means and crosstabulations were computed. Health Management Information System (HMIS) data on FP uptake were organized in an Excel spreadsheet (Office 365 v.1808 ^20^) for descriptive analysis and were represented graphically to illustrate changes over time.

***Qualitative data.*** Qualitative data gathered through IDIs with providers, community key informants and government representatives were analyzed using an applied thematic analysis approach ^21^. A team of two qualitative analysts created a structured codebook for each type of interview and tested them on the first few IDIs available for analysis, and coded all transcripts in NVivo 11 ^22^. Intercoder reliability was established at 92% ^23^. Analysis memos were developed to summarize findings related to the interview domains.

### Ethical considerations and consent

FHI 360’s Protection of Human Subjects Committee (PHSC) granted this evaluation (Project #: 1106971) research exempt status according to the requirements under 45 CFR 46.101. Burkina Faso’s Comité d’Ethique pour la Recherche Santé (CER-Ethics Committee for Health Research) in the Ministry of Health does not exempt any health research involving human subjects. CER approved the evaluation (Deliberation No. 2017-11-173) without reservations or recommendations. All participants voluntarily agreed to take part in the evaluation following the written informed consent process executed by trained data collectors.

## Results

### Family planning client characteristics and method choice

A total of 425 new FP clients were interviewed for the evaluation (see underlying data ^10^). The average age of participants was 27.8 years with women ranging from 17 to 49 years old ( Table 2). Most women were married, had not attended school and had already given birth. The average age of the youngest child was about one year old. More than 75% wanted to have a baby sometime in the future, with 57% wanting that to occur more than two years from the time they were interviewed. Another 15% wanted to give birth within the next two years.

About 47% of participants were LARC acceptors recruited from clinics and the remaining 53% were clients of CHWs who chose injectable contraception or pills. Just over 46% were using a contraceptive method for the first time. Injectable contraception and implants were used most commonly, followed by IUDs and pills ( Table 2).

**Table 2.** Sociodemographic and family planning characteristics of evaluation participants.

Demographics and family planning characteristics	Percent or mean (range, SD) (n=425)
District	
Dandé	33.9
Tougan	66.1
Age in years	27.8 (17 – 49; 7.2)
Marital status	
Single	8.0
Married	86.6
Unmarried, living together	4.5
Separated/divorced	0.7
Widowed	0.2
Highest class completed	
No school	65.4
Primary	19.8
Secondary (1 ^st^ cycle)	12.9
Secondary (2 ^nd^ cycle)	0.5
Post-secondary	0.2
Other:	1.2
Given birth to any children	95.5
Age of your youngest child in years	1.2 (0 – 11; 1.7)
Would like to have a(nother) baby sometime in the future	
No	17.7
Yes	75.5
Unsure/Don't know	6.8
When want to have first/next baby?	
In the next two years	14.8
More than two years from now	57.4
It’s not up to me	0.45
Other	0.7
Unsure/Don’t know	2.1
Client type	
Primary provider client	47.1
CHW client	52.9
First time used FP	46.6
FP method recently accepted	
Pills	4.5
Injectable	48.5
Implant	30.6
IUD	16.5

IUD – intrauterine device

With regard to the key outcome variable, 85.7% (95% CI: 82.3% to 89%) of clients reported that the provider discussed possible and normal side effects associated with use of their chosen method. This figure was significantly greater than our base estimate of 75%, which allowed us to obtain a more than adequate sample size.

### Feasibility

Feasibility of the pilot intervention was assessed by asking primary care providers and CHWs about changes in their workload, the integration of clinic activities with task sharing of IUD and implant services, and if task sharing created or exacerbated stockouts of commodities. Government officials were asked if they thought the task sharing intervention worked well, while community representatives were asked if they felt the intervention met the needs of the people.

Over half of providers interviewed stated that their workload increased as a result of participating in the pilot intervention. Among primary care providers, some described spending more time counseling clients or having more clients; some mentioned that having few providers in the health facilities increased their workload. Nevertheless, most said that they were not over-burdened by the increases and some mentioned they were gaining useful experiences through the pilot.

CHWs tended to describe larger or more burdensome increases in their workload and noted that they had responsibilities that were different from and in addition to their existing work. Others noted that they experienced challenges with transportation, accessing clients and not being paid for their work in the pilot.

*It [the pilot] has increased the workload, because we are doing two jobs. We work for the government and for task sharing. But the two jobs, it is the government that gives us [CFA] 20000 [approximately USD $35]. On the other side, we gain nothing. [CHW, Dandé]*

Regarding service integration in primary care facilities, it appeared that increasing access to LARC provision may have overwhelmed some facilities but did not affect others. Some providers stated that providing LARCs did not affect the clinics’ other activities and that they were able to provide FP methods during consultations for other issues or refer to the maternity ** unit. Others mentioned that women who came to facilities to obtain IUDs or implants during the pilot had to wait or come back when a provider was free. Some respondents said that the pilot increased wait times at the clinic.

When asked about stockouts, a few primary care providers described current or prior issues with having stockouts of implants and basic materials. A few primary care providers mentioned that women went to other facilities for FP methods due to stockouts. Only two CHWs mentioned issues with stockouts; the majority mentioned that they did not have any problems or avoided stockouts by being proactive about ordering additional supplies before they ran out.

MoH officials noted that the pilot intervention increased the availability of and demand for LARCs, and they considered that a success. One government official noted that in the regions where the program was active, there was someone capable of providing LARCs at every health center, while in the communities, there was a health worker who could offer pills and injectables. Ministry officials also noted the greatest challenge as the lack of financial incentives for providers, especially CHWs. One government official also noted that provider attrition was a problem, with trained providers leaving and being replaced with untrained counterparts.

Community representatives felt that the intervention met the needs of the people, particularly because women did not need to travel far for FP services. Community sensitization to FP that accompanied the pilot intervention was also noted as helpful, as it motivated men to get on board, which empowered women to plan their families.

*This project is thought to help our village a lot. It allowed women to have children on time. So, if the program is sustainable, you will see that there are many changes in the lives of people. [Community representative, Dandé]*

### Acceptability

***Acceptability as measured by client satisfaction.*** Client reports reflected satisfaction with services and with providers. About 84% of FP acceptors reported being very satisfied with their method (with slightly fewer IUD users very satisfied), while 12% were somewhat satisfied and less than 1% were not satisfied with their chosen method ( Table 3). With regard to satisfaction with overall services, pill users were most apt to be very satisfied, followed by injectable, implant and IUD acceptors. A similar picture emerged when clients were asked how satisfied they were with their particular provider’s services.

**Table 3.** Satisfaction indicators by method type.

Satisfaction with method	Pills % (n=19)	Injectable % (n=206)	Implant % (n=130)	IUD % (n=70)	Total % (n=425)
Very satisfied	89.5	85.9	83.9	78.6	84.2
Somewhat satisfied	10.5	9.7	13.1	17.1	12.0
Not satisfied	0.0	0.5	0.0	0.0	0.2
Don’t know/no response	0.0	2.4	2.3	4.3	2.6
Refused	0.0	1.5	0.8	0.0	0.9
**Satisfaction with services overall**					
Very satisfied	94.7	88.8	87.7	84.3	88.0
Somewhat satisfied	5.3	10.2	12.3	15.7	11.5
Not satisfied	0.0	1.0	0.0	0.0	0.5
**Satisfaction with FP provider**					
Very satisfied	94.7	93.7	94.6	88.6	93.2
Somewhat satisfied	5.3	5.8	5.4	11.4	6.6
Not satisfied	0.0	0.5	0.0	0.0	0.2

Examination of satisfaction indicators by provider type ( Table 4), indicated that the clients of CHWs and primary care providers were equally satisfied with their chosen method and overall services. However, when asked how this experience of initiating a new FP method compared with their usual experience of receiving health care services, more clients of primary care providers reported that it was a better experience than clients of CHWs (75.0% vs. 62.1%, p=0.004).

**Table 4.** Satisfaction indicators by provider type.

**Satisfaction with method**			
Very satisfied	82.0	86.2	84.2
Somewhat satisfied	14.5	9.8	0.2
Not satisfied	0.0	0.4	0.2
Don’t know/no response	3.0	2.2	2.6
Refused	0.0	0.0	0.0
**Satisfaction with services overall**			
Very satisfied	86.5	89.3	88.0
Somewhat satisfied	13.5	9.8	11.5
Not satisfied	0.0	0.9	0.5
**Satisfaction with FP provider ^1^**			
Very satisfied	92.5	93.8	93.2
Somewhat satisfied	7.5	5.8	6.6
Not satisfied	0.0	0.4	0.2
**Comparison of this FP experience with other health care services**			
Better	75.0	62.1	68.1
Same	25.0	36.2	30.9
Worse	0.0	1.8	0.9

^1^1 community health worker client refused to respond

***Community perspectives.*** Both community representatives from Dandé described positive community attitudes towards CHWs providing short acting methods. They reported that community attitudes had changed and become more positive towards FP use and that misperceptions had decreased due to the pilot.

*It's good. People in the community see this as a help to the people ... now that even primary care providers can do it, we are happy and satisfied. What we want is for them to help health workers by giving them knowledge so that the work can be done ... There are no problems. [Community representative, Dandé]*

One representative from Tougan noted that some women were hesitant because CHWs were not formal healthcare providers and recommended that they be accompanied by a formal healthcare provider to build trust in their abilities. The other representative stated that community attitudes were mixed. For example, the representative mentioned that some men are supportive while others are not, and some community members have negative attitudes towards FP in general.

*Everyone cannot agree at the same time. There are people who have understood and who give money to their wife to go and there are others too, the woman will tell him a thousand times but he will not accept. There are people like that too. So, there are Yes and there are also No. [Community Representative, Tougan]*

Nevertheless, both community representatives from Tougan agreed that there had been an increase in FP use since the beginning of the pilot.

***Provider perspectives.*** Overall, primary care providers and CHWs expressed positive views of task sharing; no participants aired negative viewpoints. Some providers stated that task sharing allows for increased accessibility of contraceptive services to women who are not located close to a clinic, leading to positive reproductive health outcomes and allows women to receive contraceptive services more quickly.

*I think it helps the community level well … In fact, because CHWs live in the community, they can reach them easily. [Primary care provider, Dandé]*

Two CHWs stated that they like that they are able to provide contraceptive services and/or that they enjoy the work that they do.

Providers were similarly satisfied with their ability to provide services in the form of FP methods and counseling without having to refer clients to another provider. Some also mentioned the satisfaction of making a positive impact on their community. Nearly all providers stated that satisfaction with their job increased due to the pilot.

### Quality of care

Quality of care assessed from the client’s perspective is fairly high ( Table 5). Over 90% of clients said that the provider spoke to her in a friendly way and over 98% of those who asked their provider questions (about 50% of clients) reported that all their questions were answered satisfactorily. Further, more than 90% of clients of both primary care providers and CHWs reported that they talked to them about all four of the contraceptive methods in question. Other methods such as condoms, emergency contraceptive pills, Standard Days Method, withdrawal, folk remedies/herbs and the lactational amenorrhea method were also mentioned by 10% or more of FP clients, but overwhelmingly, counseling was focused on injectables, pills, implants and the IUD (see underlying data).

**Table 5.** Quality of care indicators by provider type according to client recall.

	Primary care Provider % (n=200)	Community Health Worker % (n=225)	Total % (n=425)
**Provider discussed …**			
**Advantages of method**	89.0	82.2	85.4
**Disadvantages of method**	86.5	75.1	80.5
**Danger or warning signs of method**	89.5	80.0	84.5
**Possible side effects that are normal**	91.5	80.4	85.7
**Possibility of menstrual irregularities**	92.5	80.9	86.4
**What to do if experienced problems or side effects**	94.0	85.8	89.7
**Who made the decision to use the new method**			
Participant alone	78.5	92.4	85.9
Provider alone	5.5	4.0	4.7
Participant and provider together	13.5	2.7	7.8

Over three quarters of FP initiators recounted that counseling included discussion of advantages and disadvantages, danger/warning signs, possible side effects (including menstrual irregularities) and instructions on what to do if problems or side effects are experienced ( Table 5). In general, clients of CHWs were less likely than primary care clients to report that these issues were discussed. Notably, CHW clients were more likely to have made the decision to use their new method alone, while clients of primary care providers were more likely to have shared that decision with their provider.

### Safety

Very few FP clients reported any outcomes indicative of unsafe contraceptive method administration or use. All incidents of abscesses or infections were reported by injectable clients, and those events were reported by less than 1% of this group.

Health Management Information System monthly report forms recorded, among other phenomena, indicators of unsafe provision of the four FP methods under study: needle sticks to providers and to clients, complications of implant or IUD insertion, and unspecified undesirable effects associated with pill, injectable, implant or IUD use. Many more unspecified undesirable effects were reported compared to complications with implant insertion and needlestick injuries to a provider or a client ( Table 6).

**Table 6.** HMIS data on complications, undesirable effects and injuries associated with FP methods.

	Dandé		Tougan	
	CHW *	Primary **	CHW *	Primary **
IUD COMPLICATIONS	0	0	0	0
IMPLANT COMPLICATIONS	0	1	0	0
IUD UNDESIRABLE EFFECTS	0	12	0	0
IMPLANT UNDESIRABLE EFFECTS	0	16	0	1
INJECTABLE UNDESIRABLE EFFECTS	2	12	12	3
PILL UNDESIRABLE EFFECTS	0	0	1	0
NEEDLE STICK INJURIES	0	0	0	1 ***
CLIENT INJURIES	0	1	0	0

CHW – community health worker, IUD – intrauterine device * Data recorded from February to December 2017 ** Data recorded from January 2016 to December 2017 *** Recorded before provider training or pilot initiation

### Family planning uptake

**Figure 1.**
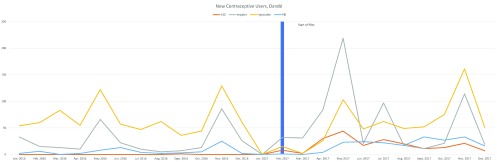
Uptake of family planning methods in Dandé, January 2016 to December 2017.

**Figure 2.**
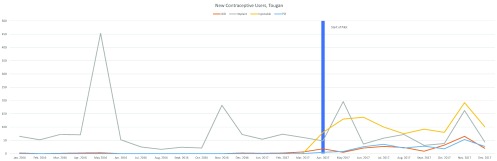
Uptake of family planning methods in Tougan, January 2016 to December 2017.

As illustrated by Figure 1 and Figure 2, there is no general pattern of overall and continuing increase in FP uptake among new acceptors after the initiation of and during the pilot intervention. This is especially evident in Tougan. However, in both Tougan and Dandé, there is a notable increase in implant uptake shortly after the initiation and during the first two to four months of the pilot intervention. These increases coincide with the two occasions in the year (May and November) that the government provides free contraception. Otherwise, the general pattern in both districts displays periodic increases (and decreases) in implant and injectable uptake among new users. The uptake among pill and IUD acceptors shows small increases since the pilot began, but it is relatively flat compared to the more popular methods of implants and injectables.

## Discussion and Recommendations

The results from this evaluation were largely positive. With regard to perceptions on feasibility, acceptability, and quality of care, the pilot intervention was a success and reinforces findings from other pilot studies of task sharing ^24, 25^. That is, injectable contraception clients, in particular, are pleased with the provision of this method through CHWs, who in turn have been shown to be capable of and amenable to providing this service. With regard to LARCs, and implants in particular, CHWs and nurses trained to provide this method demonstrated the feasibility and safety ^5^ and increased access to contraceptive methods ^26^.

This pilot intervention was unique in that several health provider cadres and methods were involved simultaneously in this task sharing enterprise, confirming that with strong stakeholder engagement and coordination, task sharing can be implemented at multiple levels simultaneously.

The providers, community representatives and government officials included in this evaluation all spoke supportively of the intervention and considered it a boon to women and the communities in which they live. FP clients were satisfied with their methods and the services they received from their respective providers. Notably, almost half of the FP clients interviewed were first time FP acceptors, suggesting that task sharing may have increased accessibility of family planning methods for new users. Both primary care providers and CHWs report that stockouts were not a major problem and the increase in their workload was largely manageable. Client reports also suggested that trained primary care providers and CHWs were safely providing FP services to their clients.

 HMIS data on pills, injectables, implants and IUDs from January 2016 to December 2017 did not show a clear and steady increase in method uptake beyond spikes coinciding with free contraceptive weeks in May and November. The data were reviewed on an ongoing basis by Consortium members during the pilot, which continued past the dates of this evaluation. Consortium members believe that while free FP days were drivers behind spikes in uptake, task sharing would have also contributed to this, and may have even helped drive the success of the free days. Our finding that about 47% of FP clients were first-time users of FP may support this assertion.

The Consortium’s discussion of the trends in uptake also resulted in recommendations to support these efforts with more effective sensitization activities and demand generation. Perhaps a longer evaluation timeframe would have detected additional changes in method uptake, but the scope of this evaluation did not include obtaining data beyond calendar year 2017. An additional limitation of the evaluation was potential bias caused by interviewing only those clients who accepted an FP method. By obtaining just the perspectives of acceptors, we may have forfeited important information on ways to improve the knowledge and practice of primary care providers and CHWs. Our goal, however, within logistical and financial constraints was to quickly identify, interview and capture the experience of an FP user who recently received services from an intervention-trained provider. Accordingly, we encourage the Consortium to seek out the perspectives of relevant non-contraceptors to obtain a complete picture that will further guide scale-up efforts.

Within the scope of this evaluation, there were important insights relevant to scale-up. For example, there were a few reports that some LARC clients were obliged to wait until a provider was available or were sent elsewhere to obtain an IUD or implant. As such, the pilot facilities may not have had the resources to meet the demand for LARC provision. Before scale-up of the intervention, a sufficient number of providers should be trained and available to insert IUDs and implants in designated facilities, especially during free contraceptive weeks in May and November. Relatedly, there was high turnover or attrition of trained providers as noted in this evaluation and mentioned elsewhere ^18^. Strategies for scale-up will need to address this phenomenon.

Secondly, quality of care--as rated by clients--was fairly high. However, when the individual indicators of quality of care were examined by provider type, there were clear differences in the information being provided to CHW clients versus the clients of primary care providers. This suggests that training and follow-up of CHWs may need to be augmented, as CHW clients were less likely to report that their provider talked to them about advantages, disadvantages, warning signs, side effects or side effects management of their chosen method. This may also explain why more primary care clients reported that they shared decision making on method choice than CHW clients: more discussion of the method and its appropriate use likely contributed to this feeling of collaboration and reiterates the wisdom of training CHWs to provide more information to clients.

Thirdly, both primary care cadres and CHWs reported job satisfaction. MoH officials remarked on the success of the training and follow-up supervision, but like providers, mentioned the lack of financial incentives as a possible impediment to increasing the reach and impact of the intervention. Although most providers stated that the increased workload created by task sharing was not a problem, CHWs in particular, were vocal about not being paid for the additional work. Given the predominance of injectables in the method mix and the importance of CHW provision, scale-up efforts should include incentives that keep CHWs engaged and an integral part of task sharing injectable contraceptive provision.

Lastly, the addition of community representative voices to the evaluation was informative. Community representatives bolstered the appeals of providers by emphasizing the importance of community sensitization with regard to generating the demand for and provision of FP. Community representatives also believed that FP uptake increased during the pilot intervention period, thus supporting perceptions of MoH officials and providers (if not the HMIS data). Community representatives can play a more sustained and systematic role by coordinating and leading sensitization events in their communities to contribute to greater male involvement in FP uptake.

Given the positive results, this evaluation demonstrated that task sharing is a feasible and acceptable approach to increasing women’s access to various FP services in this setting. Indeed, there is now a plan in place for scaling up the pilot for 2020–2022. The plan was developed and drafted in August 2019 by the pilot consortium partners, and as of December 2019, waits final validation and funding to be put into action.

## Data availability

### Underlying data

We cannot provide transcripts or summary notes of the interviews because of the very real possibility of participant identification. Quantitative data for FP clients is available via Harvard Dataverse.

Harvard Dataverse: External evaluation of a pilot intervention to increase access to family planning services in Burkina Faso. https://doi.org/10.7910/DVN/PSIK4Q ^10^

This project contains the following underlying data:

- client_deid.tab (Codebook for dataset)
- client_codebook.xlsx (Deidentified quantitative data)

### Extended data

Harvard Dataverse: External evaluation of a pilot intervention to increase access to family planning services in Burkina Faso. https://doi.org/10.7910/DVN/PSIK4Q ^10^

This project contains the following extended data:

- 1106971_Burkina_IDI_guide_provider_v1.0_clean.docx (Qualitative interview guide for Community Health Workers and Primary Care Providers)
- 1106971_Burkina_IDI_guide_gov_official_v1.0_clean.docx (Qualitative interview guide for government officials)
- 1106971_Burkina_IDI_guide_comm_rep_v1.0_clean.docx (Qualitative interview guide for Community Representatives)

Data are available under the terms of the Creative Commons Zero "No rights reserved" data waiver (CC0 1.0 Public domain dedication).

## Footnotes

^a^ A total of 8 MoH officials were initially identified for key informant interviews, but we were unable to conduct the eighth IDI within a reasonable amount of time.
