# Supplementary material for: Evaluation of a pilot program for task sharing short and long-acting contraceptive methods in Burkina Faso
Source: Gates Open Res. 2020 Feb 11;3:1499. Originally published 2019 Jun 25. [Version 2] doi: 10.12688/gatesopenres.13009.2 (PMC7029614; doi:10.12688/gatesopenres.13009.2)
Supplement: Peer review history

# Peer review: Reviewer response for version 2

**DOI:** 10.21956/gatesopenres.14280.r28574

**Published:** 2020-03-03

**Authors:** Eileen A. Yam

**Affiliations:** 1 Population Council, Washington, DC, USA

The authors have satisfactorily addressed my comments on Version 1.

I confirm that I have read this submission and believe that I have an appropriate level of expertise to confirm that it is of an acceptable scientific standard.

# Peer review: Reviewer response for version 2

**DOI:** 10.21956/gatesopenres.14280.r28572

**Published:** 2020-02-18

**Authors:** Sarah Saleem, Saleem Jessani

**Affiliations:** 1 Department of Community Health Sciences, The Aga Khan University, Karachi, Pakistan

I am satisfied with the changes incorporated by the authors, and I have no further comments to make.

We confirm that we have read this submission and believe that we have an appropriate level of expertise to confirm that it is of an acceptable scientific standard.

# Peer review: Reviewer response for version 1

**DOI:** 10.21956/gatesopenres.14117.r27448

**Published:** 2019-09-09

**Authors:** Clémentine Rossier

**Affiliations:** 1 Institut de démographie et socioéconomie, University of Geneva, Geneva, Switzerland

The paper is well written, task-sharing is an important programmatic step to expand contraceptive services in low income countries, and a pilot study on a country such as BF where task-sharing is not yet a national policy is an important endeavour. Methods and analysis are sound as well.

 This paper needs to be strengthened along several lines however. First a theoretical section summarizing the literature on task-sharing is missing: what effect on uptake was found in other sites? What obstacles and challenges have been documented in studies so far? In particular, only if all existing studies have demonstrated an effect of task sharing on contraceptive uptake can the following sentence be introduced, and only after mentioning these studies: “Burkina Faso can truly accelerate FP uptake by promoting measures such as task sharing that expand access to FP services”. This should be stated as a question, one of the question this pilot study attempts to answer.

 The originality of the project should be stated right after this review of the literature. The following sentence comes much too late: “This project is unique in that several health provider cadres and methods were involved simultaneously in this task sharing enterprise, confirming that with strong stakeholder engagement and coordination, task sharing can be implemented at multiple levels simultaneously.”

 Altogether, the first part of the paper (introduction and results) sounds biased in favour of the intervention, because possible problems are not mentioned in the introduction, and because the effect of the intervention on uptake is not introduced as an open question. Results seem to devote too much space to positive aspects. For example, should so much space in the results section be devoted to informants stating that FP use has increased? Service statistics shows that this is an impression, probably linked to the occurrence of free contraceptive weeks, but that no clear link could be made with the introduction of task sharing.

 In the discussion the authors should state more clearly that the pilot did not increase FP uptake, and discuss this in the context of free contraceptive weeks (i.e. this other intervention may have increased demand to its maximum, so that additional task-sharing did not change anything; task-sharing should be introduced in a controlled environment, where no other interventions are on-going, to show its effects on demand). Other than that, the discussion is more balanced and critical and thus useful; the introduction and results should be written in this scientific tone.

I confirm that I have read this submission and believe that I have an appropriate level of expertise to confirm that it is of an acceptable scientific standard, however I have significant reservations, as outlined above.

# Response

**Published:** 2020-02-03

**Authors:** Dawn Chin-Quee

**Evaluation of a pilot program for task sharing short and long-acting contraceptive methods in Burkina Faso: Response to Reviewers**

Reviewer 3: Clementine Rossier

The authors of the above-titled manuscript express their gratitude to the reviewers who provided very helpful feedback—most of which was incorporated into Version 2 of the article. Below, the first author responds directly to the comments and suggestions proffered by Reviewer 3.

**Background/Introduction**

1. Reviewer 3 mentioned that the Introduction needed background information on task sharing and its known impact as well as more details on the current situation in Burkina Faso and the problem statement as it relates to task sharing. We have expanded the Introduction to include this information.

**Results**

1. With additional information provided on the intervention and reactions to low FP uptake, we hope a more balanced picture of the results has been provided. There are positive findings (made more captivating by participants’ quotes), but there are also negative ones that should not get lost, even with qualitative findings that typically add salient and compelling texture to research. For example, we report that wait times increased for FP services in health centers due to the intervention; CHWs felt taken advantage of by not being paid for the pilot; clients of CHWs reported less satisfaction than their counterparts who received services from primary care cadres, provider attrition occurred throughout the intervention; community representatives reported mixed or negative attitudes towards FP (often expressed by men); and of course, FP uptake was dismal. We are glad that the Discussion is considered more balanced, critical and useful.

**Discussion and Recommendations**

1. We prefer to keep our sentence on the originality of the project (“This project is unique in that several health provider cadres and methods were involved simultaneously in this task sharing enterprise, confirming that with strong stakeholder engagement and coordination, task sharing can be implemented at multiple levels simultaneously”) in the Discussion section, where we consider it to be a strength. In addition, because the second part of the sentence refers to results/outcomes, we feel it would be out-of-place in the Introduction.

# Peer review: Reviewer response for version 1

**DOI:** 10.21956/gatesopenres.14117.r27445

**Published:** 2019-07-17

**Authors:** Sarah Saleem, Saleem Jessani

**Affiliations:** 1 Department of Community Health Sciences, The Aga Khan University, Karachi, Pakistan

The paper “Evaluation of a pilot program for task sharing short and long-acting contraceptive methods in Burkina Faso” by Quee at al. is an interesting and detailed program evaluation report of a pilot study conducted in Burkina Faso which aimed to “increasing access to family planning (FP) services through task-sharing for short- and long-acting family planning methods to primary care cadres.” However, the following points should be addressed for strengthening the paper in order to convey a clear message to readers.

 Abstract:

1. Background: It should be limited to program background and objective only. The details related trainings can be moved to methodology.
2. Methodology: It describes about the type of tools used for quantitative and qualitative components However, sample sizes are missing. Moreover, some details of service statistics are also required.
3. Conclusion: It is a very general statement and should be based on the results presented providing a way forward.

Introduction:

1. Task sharing has been justified, however there is no information about the health system in Burkina Faso describing the need of task sharing.
2. Details of the pilot project including the indicators reported for data collection, analyses and interpretation can be moved to methodology.

Methods:

1. It would be good if 2 ^nd^ paragraph on page 4 “Indepth interviews (IDIs) ……….” along with the sample size and the details of service statistics include information on what statistics have been used for evaluating the program.
2. For sample size, authors have used an assumption that at least 75% providers will talk about side effects of contraceptive methods. It would be good if reference is provided for this assumption.
3. There should be an explicit note of eligibility criteria of participants enrolled in various assessments.
4. There are chances of selection bias with inclusion of new FP users only in the quantitative assessment as non-acceptors were not interviewed. This limitation can be addressed in the discussion section
5. In the description of pilot intervention, it has been mentioned that events that sensitized the community to FP were held but there are no further details. The intervention should be spelled out in methodology clearly.

Results:

1. The results showed that 425 new FP clients were interviewed; however, it would be good if authors can report response rate, i.e. how many of them refused to participate in the study, and how many of them actually were in the records.   
2. During the intervention period, how many participants were approached; of these how many accepted FP method and how many were the continued uses at the time of interviews.
3. There should be some comparative analysis supported by relevant statistical tests. 

Discussion:

1. Although, the results from the qualitative component were most addressed, however, HMIS data before and after intervention were inconclusive. Therefore, results should be interpreted cautiously.
2. Strengths and limitations of the study are missing. 

Overall impression: The article can be indexed after suggested revisions.

We confirm that we have read this submission and believe that we have an appropriate level of expertise to confirm that it is of an acceptable scientific standard, however we have significant reservations, as outlined above.

# Response

**Published:** 2020-02-03

**Authors:** Dawn Chin-Quee

**Evaluation of a pilot program for task sharing short and long-acting contraceptive methods in Burkina Faso: Response to Reviewers**

Reviewer 2: Sarah Saleem, Saleem Jessani

The authors of the above-titled manuscript express their gratitude to the reviewers who provided very helpful feedback—most of which was incorporated into Version 2 of the article. Below, the first author responds directly to the comments and suggestions proffered by the Reviewer 2.

**Abstract**

1. Based on comments from Reviewer 2, we have included sample sizes and provided more information on service statistics. We also updated the Conclusion to mention how the results guided decisions/actions for the way forward.
2. We have removed references to “training” in the abstract, as advised by Reviewer 2, which now describes the program background re: the task sharing activities of the 4 provider cadres.

**Background/Introduction**

1. Reviewer 2 mentioned that the Introduction needed more details on the current situation in Burkina Faso and a problem statement. We have expanded the Introduction to include this information.
2. As Reviewer 2 pointed out, the discussion of evaluation indicators is better placed in the Methods section. They have been moved to that section. However, we feel that the description of the pilot study belongs in the Introduction and not in Methods, as it provides the background for the evaluation and how we operationalized said evaluation in the Methods section.

**Methods**

1. We have now provided sample sizes and details on the statistics that were performed on service statistics.
2. We have now specified that our assumption that at least 75% of providers will talk about side effects (after being newly trained) was based on experience. Our assumption was a good one, as our results showed that more than 75% did do so.
3. We have added text that explicitly states eligibility for FP clients. For providers, it is mentioned that they are a subset of those trained for the pilot program, and key informants (MoH officials and community representatives) were purposively selected as described in the text.
4. A revision of the description of the pilot intervention (in the Introduction) now provides more information on the intervention.
5. Reviewer 2 pointed out a limitation that we did not address. We have now included in the Discussion section that we may have been subject to selection bias, since all the client exit interviews we conducted with were with FP acceptors.

**Results**

1. We do not have information on response rates, as only FP clients who indicated to providers that they were interested in participating in the evaluation were then sent on to or contacted by RAs for interviews. That is, we did not charge providers with collecting response rates. Also, the women we recruited were very recent acceptors of the FP methods under study (no more than 1 week), so, there would be no value in examining continued use by the time of exit interviews. Finally, we did not conduct comparative analysis as that was outside the scope of the evaluation.

**Discussion and Recommendations**

1. We have not explicitly laid out subheadings in the Discussion section with “Strengths” and “Limitations”, but consider the revised text clear on what should be considered strengths and limitations of the evaluation.

# Peer review: Reviewer response for version 1

**DOI:** 10.21956/gatesopenres.14117.r27446

**Published:** 2019-06-28

**Authors:** Eileen A. Yam

**Affiliations:** 1 Population Council, Washington, DC, USA

This paper is a largely descriptive analysis of a pilot program to task share provision of short- and long-acting family planning with primary care providers and community health workers in two districts in Burkina Faso. The substantive take-aways are clear and substantiated by presented findings, and most of my suggestions are to clarify or strengthen the language. The qualitative data cannot be shared, so understandably, those source data are not available. 

 Abstract

1. Methods: what kind of service statistics, and how many exit interviews and IDIs?
2. The Conclusions are very thin and not particularly informative. What kinds of decisions can be guided by these findings? 

Introduction

1. There seems to be a missing problem statement. What are the challenges that task sharing can address? For instance, can you cite data on whatever the problems are in Burkina Faso, like the shortage of specialists, geographic disparities in service coverage, overburdened doctors, etc.?
2. Who are these individuals referred to as "primary care cadres?" The description of who they are is in the next paragraph, but it makes more sense to shift those definitions here, since you first mention these cadres here. 
3. What sectors are these 26 health centers? All public? How did these cadres identify which patients were to be offered FP services, since the sites were primary care services as opposed to dedicated FP sites?
4. The list of indicators of interest are better placed in the Methods section describing the analysis, rather than the Introduction. Also, are the "reports of client satisfaction" the reports of women, or of providers?

Method

1. Since these are primary care services, what was the standard operating procedure for identifying and providing FP services to the subset of patients who want FP? Presumably, many/most primary care patients are not actually looking for FP services.
2. For the qualitative component, can you provide any more information on the four community representatives? Can you at least describe the genders of these individuals?
3. On p. 5, in the paragraph describing ethical review, the text seems to suggest that the protocol received a non-research determination from FHI 360's ethical review board (i.e., exempt), but that it was not exempt by the in-country ethical review boards. Why the discrepant determinations?

Table 1

- Add some brief language describing what registered birth attendants, auxiliary birth attenants, and "mobile" health workers are.

Discussion and Recommendations

- Only FP acceptors are interviewed, so a potential limitation is that the study team did not get the perspectives of those who may not have opted to accept FP, despite having been counseled by one of the providers in the pilot. Isn't it possible that women were counseled by these providers, but perhaps did not have a good experience and, therefore, did not accept FP? If the specifics of the intervention were spelled out more clearly up front in the description of the pilot, the answer to this question may be evident.

I confirm that I have read this submission and believe that I have an appropriate level of expertise to confirm that it is of an acceptable scientific standard, however I have significant reservations, as outlined above.

# Response

**Published:** 2020-02-03

**Authors:** Dawn Chin-Quee

**Evaluation of a pilot program for task sharing short and long-acting contraceptive methods in Burkina Faso: Response to Reviewers**

**Reviewer 1:** Eileen Yam

The authors of the above-titled manuscript express their gratitude to the reviewers who provided very helpful feedback—most of which was incorporated into Version 2 of the article. Below, the first author responds directly to the comments and suggestions proffered by Reviewer 1.

**Abstract**

1. We have included sample sizes and provided more information on service statistics. We also updated the Conclusion to mention how the results guided decisions/actions for the way forward.

**Background/Introduction**

1. Reviewer 1 mentioned that the Introduction needed background information on task sharing, more details on the current situation in Burkina Faso and the problem statement as it relates to task sharing. We have expanded the Introduction to include this information.
2. As Reviewer 1 pointed out, the discussion of evaluation indicators is better placed in the Methods section. They have been moved to that section.
3. We now describe Primary Care Cadres in the same paragraph when they are first mentioned (no need to footnote in Table 1).
4. We have specified that the 26 health centers are public/government entities and that client satisfaction is based on the reports of FP clients themselves. We assume that all participants voluntarily made their interest in FP services known to primary cadres, so there would be no need for them to identify potential acceptors specifically for the evaluation of the pilot. With regard to the evaluation itself, research assistants (RAs) knew which clients to approach for client exit interviews, because the primary care cadres had already served them as FP clients and could ascertain their interest in participation before sending them over (IUD, implant acceptors) or providing contact information (pills, injectable acceptors) to the RA to arrange for an interview.

**Methods**

1. We cannot provide any more demographic information on the 4 community representatives, not even gender, as their numbers are so small and they are known for the role they play in the community. We deliberately collected information just on location.
2. We have now specified in the text that Burkina Faso’s IRB, CER does not exempt any study involving human subjects.

**Results**

1. Since we have described the primary cadres in the Introductory text, we will not include a footnote of the same for Table 1.

**Discussion and Recommendations**

1. Reviewer 1 (in her comments on Discussion and Recommendations) pointed out a limitation that we did not address. We have now included in the Discussion section that we may have been subject to selection bias, since all the client exit interviews we conducted with were with FP acceptors.

 

**Evaluation of a pilot program for task sharing short and long-acting contraceptive methods in Burkina Faso: Response to Reviewers**

**Reviewer 1:** Eileen Yam

The authors of the above-titled manuscript express their gratitude to the reviewers who provided very helpful feedback—most of which was incorporated into Version 2 of the article. Below, the first author responds directly to the comments and suggestions proffered by Reviewer 1.

**Abstract**

1. We have included sample sizes and provided more information on service statistics. We also updated the Conclusion to mention how the results guided decisions/actions for the way forward.

**Background/Introduction**

1. Reviewer 1 mentioned that the Introduction needed background information on task sharing, more details on the current situation in Burkina Faso and the problem statement as it relates to task sharing. We have expanded the Introduction to include this information.
2. As Reviewer 1 pointed out, the discussion of evaluation indicators is better placed in the Methods section. They have been moved to that section.
3. We now describe Primary Care Cadres in the same paragraph when they are first mentioned (no need to footnote in Table 1).
4. We have specified that the 26 health centers are public/government entities and that client satisfaction is based on the reports of FP clients themselves. We assume that all participants voluntarily made their interest in FP services known to primary cadres, so there would be no need for them to identify potential acceptors specifically for the evaluation of the pilot. With regard to the evaluation itself, research assistants (RAs) knew which clients to approach for client exit interviews, because the primary care cadres had already served them as FP clients and could ascertain their interest in participation before sending them over (IUD, implant acceptors) or providing contact information (pills, injectable acceptors) to the RA to arrange for an interview.

**Methods**

1. We cannot provide any more demographic information on the 4 community representatives, not even gender, as their numbers are so small and they are known for the role they play in the community. We deliberately collected information just on location.
2. We have now specified in the text that Burkina Faso’s IRB, CER does not exempt any study involving human subjects.

**Results**

1. Since we have described the primary cadres in the Introductory text, we will not include a footnote of the same for Table 1.

**Discussion and Recommendations**

1. Reviewer 1 (in her comments on Discussion and Recommendations) pointed out a limitation that we did not address. We have now included in the Discussion section that we may have been subject to selection bias, since all the client exit interviews we conducted with were with FP acceptors.
